# HR-MAS NMR Based Quantitative Metabolomics in Breast Cancer

**DOI:** 10.3390/metabo9020019

**Published:** 2019-01-22

**Authors:** Mikheil Gogiashvili, Jessica Nowacki, Roland Hergenröder, Jan G. Hengstler, Jörg Lambert, Karolina Edlund

**Affiliations:** 1 Leibniz Institut für Analytische Wissenschaften, ISAS, e.V., 44139 Dortmund, Germany; jessica.nowacki@tu-dortmund.de (J.N.); roland.hergenroeder@isas.de (R.H.); joerg.lambert@isas.de (J.L.); 2 Leibniz Research Centre for Working Environment and Human Factors (IfADo), 44139 Dortmund, Germany; Hengstler@ifado.de (J.G.H.); Edlund@ifado.de (K.E.)

**Keywords:** NMR, HR MAS, breast cancer, metabolomics

## Abstract

High resolution magic-angle spinning (HR-MAS) nuclear magnetic resonance (NMR) spectroscopy is increasingly used for profiling of breast cancer tissue, delivering quantitative information for approximately 40 metabolites. One unique advantage of the method is that it can be used to analyse intact tissue, thereby requiring only minimal sample preparation. Importantly, since the method is non-destructive, it allows further investigations of the same specimen using for instance transcriptomics. Here, we discuss technical aspects critical for a successful analysis—including sample handling, measurement conditions, pulse sequences for one- and two dimensional analysis, and quantification methods—and summarize available studies, with a focus on significant associations of metabolite levels with clinically relevant parameters.

## 1. Introduction 

The improved understanding of breast cancer has been supported by the development of omics-based technologies. Transcriptomics has made key contributions, for instance by delineating clinically relevant subtypes based on gene expression patterns [1,2,3]. Moreover, gene expression-based assays are now used for the assessment of recurrence risk in the clinical setting [4,5,6]. As a relative newcomer to the omics field, metabolomics offers the potential to further reveal alterations that underlie breast cancer development and progression, as well as the discovery of novel therapeutic targets and biomarkers for improved diagnostics and prediction of prognosis as well as response to therapy.

Nuclear magnetic resonance (NMR) spectroscopy and mass spectrometry (MS) are the two most widely used methods for quantitative metabolomic analysis of tumour tissue. Their respective advantages and disadvantages have been extensively reviewed elsewhere, for example, by Wishart et al. [7] and Nagana Gowda & Raftery [8]. Importantly, the higher sensitivity of MS allows the quantification of a much larger number of metabolites compared to NMR. However, despite this disadvantage of NMR spectroscopy, certain characteristics make this method indispensable in metabolomics. Properties of NMR spectroscopy, such as the capacity to provide information about the number of chemically identical atoms, the chemical shift of individual atomic groups and the spin-spin coupling with the resulting signal splitting pattern contribute to the high selectivity of this method [9]. Consequently, one-dimensional NMR spectroscopy can often be sufficient, without a need of using two dimensional (2D) NMR, to perform the reliable identification and also reproducible quantification of small molecules. Moreover, NMR does not require chemical separation of analytes prior to analysis, which is one of the reasons for its excellent technical reproducibility [8]. A particular advantage of NMR spectroscopy is the possibility to directly analyse intact tissue in a non-destructive manner, while MS requires an extraction step, thus destroying tissue integrity. Most studies use the ^1^H nucleus for sensitivity reasons. For the same reason, techniques employing the ^13^C nucleus have not found wide applications in tissue analysis and metabolomics studies that use ^13^C usually require labelled samples. ^31^P HR-MAS NMR, however, is a valuable technique for the analysis of tissue specimens, as the sensitivity is just one order of magnitude lower than that of ^1^H NMR and potential applications to both phospholipid and energy metabolism studies are evident.

**Figure 1:**
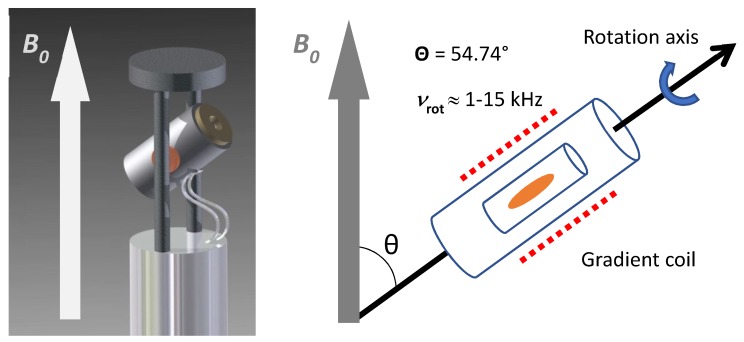
Representation of an HR-MAS NMR probe head with an orientation of the stator at an angle (θ) of 54.7° between axis of rotation and B_0_. The rotation speed (v_rot_) of the sample reaches up to 15 kHz. A gradient coil is arranged around the rotor.

Magnetic resonance spectroscopy (MRS) provides spatially resolved information about the chemical composition of tissue in vivo [10]. If combined with ex vivo NMR studies, in vivo MRS can be used to non-invasively detect biomarkers that were identified in previous ex vivo NMR studies. A disadvantage of in vivo MRS, however, is its poor spectral resolution. The ex vivo analysis of intact tissue specimens also suffers from poor resolution compared to conventional liquid NMR techniques. In liquid samples, the dipole-dipole couplings, that is, the through-space interactions between protons, as well as the anisotropy, that is, the orientational dependence of the chemical shift, are completely averaged out due to the high molecular mobility. Under these conditions, only the isotropic chemical shift and the coupling through bonds remain, giving rise to well-resolved signals with small (1–2 Hz) linewidths. For ^1^H-NMR investigations of tissue, the anisotropy of the chemical shift can be neglected but the dipole-dipole coupling, albeit small (20–50 Hz) is non-negligible [11]. Since the limited mobility of molecules in tissue impedes a full averaging of the dipolar couplings, the resulting spectra are poorly resolved. This makes quantification difficult, as the superposition of numerous broad signals from a mixture of metabolites can no longer be easily disentangled. Moreover, the semisolid character of tissue gives rise to local magnetic field gradients, caused by spatial variations of the magnetic bulk susceptibility, which also leads to spectral broadening [12,13]. Both the anisotropic part of the latter interaction [11,13] and the dipole-dipole coupling follow a [3cos^2^(Θ)-1] dependence on the angle Θ between the B_0_ field and the distance vector between interacting nuclei [11]. If the sample is rotated at the so-called "magic angle" of Θ = 54.7°, the [3cos^2^(Θ) - 1] term becomes zero and a high-resolution NMR spectrum is obtained [14,15] (Figure 1).

One of the earliest applications of high-resolution magic angle spinning (HR-MAS) NMR spectroscopy was the study of polymer beads, which are synthetic organic materials used in the preparation of combinatorial chemistry libraries [16]. Towards the end of the 1990s, the examination of intact tissue became possible [17,18,19,20], enabled by the development of high-field magnets, improved probe heads with ultra-low-inductance decoupling coils and symmetric spinner drive designs that led to a reduction of spinning-, decoupling- and variable temperature-induced thermal gradients [21,22]. The alignment of the z-axis gradient along the magic angle gave access to a wide variety of experimental NMR techniques, such as gradient enhanced solvent suppression and to the full range of 1D and 2D homo- and hetero-nuclear experiments. In this way, HR-MAS NMR spectra, with a typical linewidth of 1–2 Hz, became comparable in quality to spectra from liquid samples [23,24]. As already mentioned, HR-MAS NMR allows metabolite quantification directly from intact tissue specimens, which abolishes the need for an extraction step and thereby avoids one potential source of poor reproducibility. In addition, the risk of partial extraction of certain metabolites, as for instance reported for choline [19], is minimized. Also, the non-destructive nature of the method allows the analysis of metabolite levels in tissue to be combined with other analytical techniques based on the same tissue specimen, which may be important when tissue availability is limited.

HR-MAS NMR has been used to study metabolites in breast cancer tissue and to correlate metabolite concentrations with clinically relevant parameters. Here, we summarize the HR-MAS NMR-based studies of breast cancer tissue available until December 2018, focusing on analytical aspects, including measurement conditions, pulse sequences used for 1D and 2D NMR, quantification methods and the numbers and identities of the reported metabolites. Moreover, we summarize findings based on quantitative HR-MAS NMR with regard to significant associations with clinically relevant factors in breast cancer.

## 2. Preanalytical Factors and Measurement Conditions

**Figure 2:**
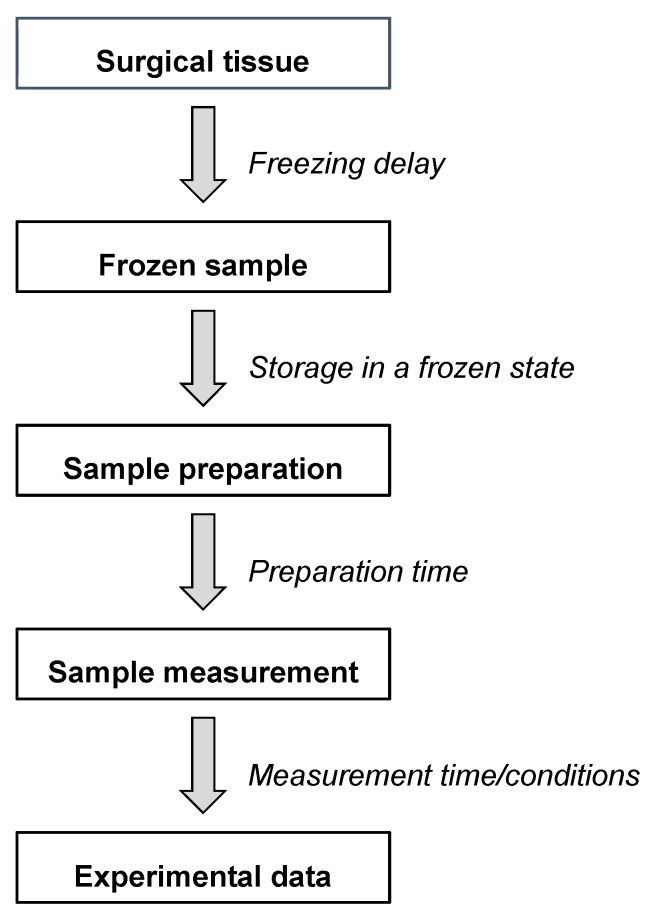
Different steps and time windows between the tissue sampling and the final HR-MAS NMR data.

Reliable and reproducible analysis of tissue samples using HR-MAS NMR requires a robust and standardized protocol that considers factors both before and during the measurement. In order to standardize the analytical conditions, various time windows should be considered, including sampling, storage, sample preparation and measurement (Figure 2). A brief overview of potential issues related with the analysis of tissue specimens using HR-MAS NMR was recently published by Esteve and colleagues [25]. However, as of yet, only few studies directly examined the impact of time and temperature during the different steps of the analytical pipeline in the analysis of breast tumour tissue [26,27].

Any omics analysis represents a snapshot of the tissue at one point in time. The time delay after the tissue has been removed from the circulation during surgery until freezing should therefore be kept as short as possible or ongoing biochemical processes in the tissue may alter the metabolite content. Haukaas et al. studied the influence of the length of the time interval before the tissue was snap-frozen in liquid nitrogen (0, 15, 30, 60, 90 and 120 min) on the metabolic profile of breast cancer xenografts [27]. A significant difference between metabolite levels in samples frozen directly after surgical removal and frozen after 60 min was shown for ascorbate (−25%), choline (+56%) and creatine (−28%) and after 90 min for glutathione (−35%). The freezing delay did not have a statistically significant influence on the eleven additionally studied metabolites but ranged from –15% to +11% and −20% to +31% after 30 and 60 min, respectively. Notably, for all time points, increased metabolite levels were observed for certain metabolites, while the levels of other metabolites decreased. Moreover, the pattern across the increasing time delays varied for the individual metabolites, including consistently increasing or decreasing metabolite levels, as well as more difficult to interpret mixed patterns. Nevertheless, no significant alterations in metabolite levels are expected at freezing delay times less than 30 min. The impact of the duration of the ischemic period before freezing was also studied in other tissue types. For instance, in rat brain, statistically significant changes were observed for glucose (down) as well as alanine, γ-aminobutyric acid (GABA) and lactate (up) after 30 min and additionally for glutamine, myo-inositol, GPC and total choline (down) and acetate, creatine and glycine (up) after three hours [28]. We are unaware of any study that directly compares the effect of ischemia/freezing delay time between different tissue types, but the occurrence of tissue-specific differences can probably not be excluded.

In the above-mentioned study by Haukaas et al., the authors also compared metabolite levels between samples that were immediately snap-frozen and thereafter analysed (*n* = 6) and samples that were analysed directly, without being frozen (*n* = 6) [27]. Importantly, significantly higher mean metabolite levels were observed for 12 of 16 metabolites in the snap-frozen tissues (20–60% increase after freezing compared to fresh tissue). Noticeable differences could also be observed between the metabolites with regard to the level of variability between the replicates (20–100%). This study is in agreement with the results of an early study of rat kidney from 1998, where considerably increased signal intensities were observed after freezing for several metabolites, including alanine (> 100%), glutamine (> 40%) and glycine (> 100%) [29]. Middleton et al. assigned these changes to the release of metabolites that were bound to macromolecules and therefore are invisible for HR-MAS NMR. This can happen due to freezing-induced cellular disruption and/or the precipitation of non-freezing resistant proteins, in both cases leading to fewer available non-specific binding sites for small molecules. Increased levels of several metabolites in rat kidney after snap-freezing were also observed by Waters et al., including leucine, isoleucine, valine, alanine and glycine, when compared to fresh tissue that was kept on ice for up to five hours before analysis [30]. In addition, decreased signals of choline, glycerophosphocholine, glucose, myo-inositol, trimethylamine N-oxide (TMAO) and taurine were found after snap-freezing. However, the statistical significance and magnitude of these changes were not reported. Interestingly, much fewer changes were observed in liver compared to kidney, indicating tissue-specific differences [30]. All in all, despite the observed freezing-induced changes, freezing the tissue is likely to remain a standard approach for practical reasons, such as the distance between the surgical unit and the laboratory, as well as programs for prospective tissue collection and biobanking for later analysis.

After snap-freezing, the storage of biological material at −80 °C until analysis is standard [31]. Only one published study thus far investigated the impact of storage time at −80 °C on the metabolic profile of human breast cancer tissue [26]. In this study, samples were snap-frozen after being kept for approximately 30 min on ice and analysed using HR-MAS NMR after 1, 6 and 12 months. It was reported that the levels of choline in healthy breast tissue increased (*p* < 0.000001) with longer storage time, while phosphocholine decreased (*p* < 0.000001), which could be due to the breakdown of phosphocholine to choline. Lower phosphocholine levels were also observed in breast tumour tissue (*p* < 0.0002), together with increased levels of lactate (*p* < 0.05). The concentrations of nine other metabolites showed no significant changes during the one year storage period. Further studies would be required to assess the impact of storage at −80 °C for even longer time periods, which usually is the case when studying, for instance, the association of metabolite concentrations with cancer survival in retrospective frozen tissue collections. Findings by Jordan et al. of no significant storage time-associated effect on metabolite levels, evaluating human prostate cancer tissue after three years of storage at −80 °C [32], support the conclusion that the influence of the storage time in a low-temperature freezer is most likely minor.

Before HR-MAS NMR, preparation of the sample for analysis includes punching or cutting the tissue to fit into an insert, placing it in the rotor, weighing, and adding the internal standard. These preparatory steps are commonly performed at room temperature, with the specimen kept on ice to avoid extensive thawing. The usage of a cooled workstation has also been reported [33,34]. Another option is to prepare the sample at −10 °C in a closed glovebox under nitrogen atmosphere [35,36]. This would, in addition, mitigate the possible influence of condensation of ambient water from the air, which may distort the sample weight and in turn affect the quantification. However, the impact of factors during the sample preparation step on final metabolite concentrations has not been systematically studied until now.

Finally, different conditions during the measurement, with regard to temperature, analysis time and rotation frequency, were used in the thus far published HR-MAS NMR studies of breast cancer tissue. To avoid line broadening and to achieve a high resolution MAS NMR spectrum, the sample must be thawed and the measurement performed at a temperature above 0 °C. To minimize the risk of temperature-induced changes during the measurement, the temperature after methanol calibration is usually adjusted to approximately 5 °C. In brain tissue, it was shown that the rate of degradation of N-acetyl aspartate (NAA) to acetate was four times higher at 20 °C than at 2 °C [19]. Most measurements of breast cancer tissue reported in the literature were performed at 4 °C [37,38,39,40,41], while others were performed at 5 °C [27,42,43,44,45], 6 °C [41,46], 19 °C [47,48], 20 °C [49] or 26 °C [50], with an approximate mean measurement time of 19 min (range: 3 min 7 sec [27] to 1 h 5 min [51]). Haukaas et al. reported that a prolonged HR-MAS NMR measurement time of 1.5 h at 5 °C influenced the concentration of certain metabolites, including a significant increase of glucose, glycine and choline and a decrease of glycerophosphocholine (GPC) [27]. Similar observations were made in brain tissue, with decreasing levels of N-acetyl aspartate (NAA) and increasing levels of acetate in spectra collected at 20 °C during the course of 24 h [19]. Similarly as for the freezing delay time, different tolerance to thawing, as well as multiple freeze-thaw cycles, can probably be expected in various tissue types, so it is difficult to assess to what extent findings in one tissue type can be extrapolated to other. In studies such as those above, it is also difficult to separate the effect of the extended time at a temperature above 0 °C from that of prolonged rotation at a high frequency. Indeed, the rotation of tissue at a high frequency during HR-MAS NMR has been reported to impact the morphology of the specimen [38,41,52] as well as the metabolite content [28]. A high speed of rotation can destroy cell and tissue structures. For instance, analysing cells, two hours of rotation at 2.5 kHz destroyed approximately 20% of adipocytes [53] and cell lysis was observed in erythrocytes at 4 kHz MAS rotation [54]. In human prostate tissue, distortion of ductal structures occurred after one hour spinning at 3 kHz; whereas, no obvious morphological alterations were observed after 45 min spinning at 600 Hz followed by 15 min at 700 Hz [52], indicating that preservation of tissue integrity could be achieved by slower rotation. However, the centrifugal forces are only reduced by two orders of magnitude when the spin rate is reduced by a factor of ten [55]. The results of one study of rat brain tissue suggest that mechanical stress due to prolonged spinning at 4 °C may have a larger impact on the metabolic profile than the delay in the freezing of the sampled tissue sample in liquid nitrogen, for instance leading to increased creatine levels, possibly because of the tissue-damage associated release from initially undetectable creatine stores [28]. In the published studies of breast cancer tissue using HR-MAS NMR, a rotation frequency of 5 kHz was applied, except in some publications spinning the sample at 2 kHz [47,48,50,51,56], 2.5 kHz [49] and 6 kHz [24]. As of yet, no study directly compared the impact of rotation frequency on tissue morphology and metabolite concentrations in breast cancer tissue. Renault et al. compared HR-MAS measurements of liver tissue at different rotations frequencies (150 Hz, 500 Hz, 4000 Hz) and found that the presence and intensity of sidebands is strongly dependent on the sample preparation (position and shape of the sample, presence of air bubbles) [57]. The authors claim that sideband free spectra can be obtained at rotation frequencies as low as 500 Hz by minimizing the volume of the sample chamber and by positioning the sample chamber at the coil centre with an insert located at the top of the rotor.

In summary, further studies are warranted to better understand the impact of conditions during sample procurement, storage and analysis of breast cancer tissue on metabolite levels analysed by HR-MAS NMR. Only one study of breast cancer tissue comprehensively examined the impact of the freezing delay time after surgery on metabolite concentrations. Given the practical difficulties of tissue collection immediately after surgery, further validation that no significant changes in metabolite concentrations occur during the first 30–60 min would be important to provide additional confidence in current protocols for tissue procurement. The impact of the rotation frequency and measurement time on metabolite concentrations have not yet been systematically examined in breast cancer tissue and should be addressed in future studies.

## 3. NMR Techniques Employed in Tissue Analysis

Several common NMR techniques can be employed in the analysis of intact tissue specimens to provide specific information about the metabolite and lipid content. A successful analysis also depends on techniques to suppress unwanted signals that originate from, for instance, tissue water and the macromolecular background of lipids and proteins that would otherwise impede the analysis. Below, the presaturation approach to suppress solvent signals, as well as common pulse sequences used for one and two dimensional NMR experiments, are described.

### 3.1. Water Suppression

**Figure 3:**
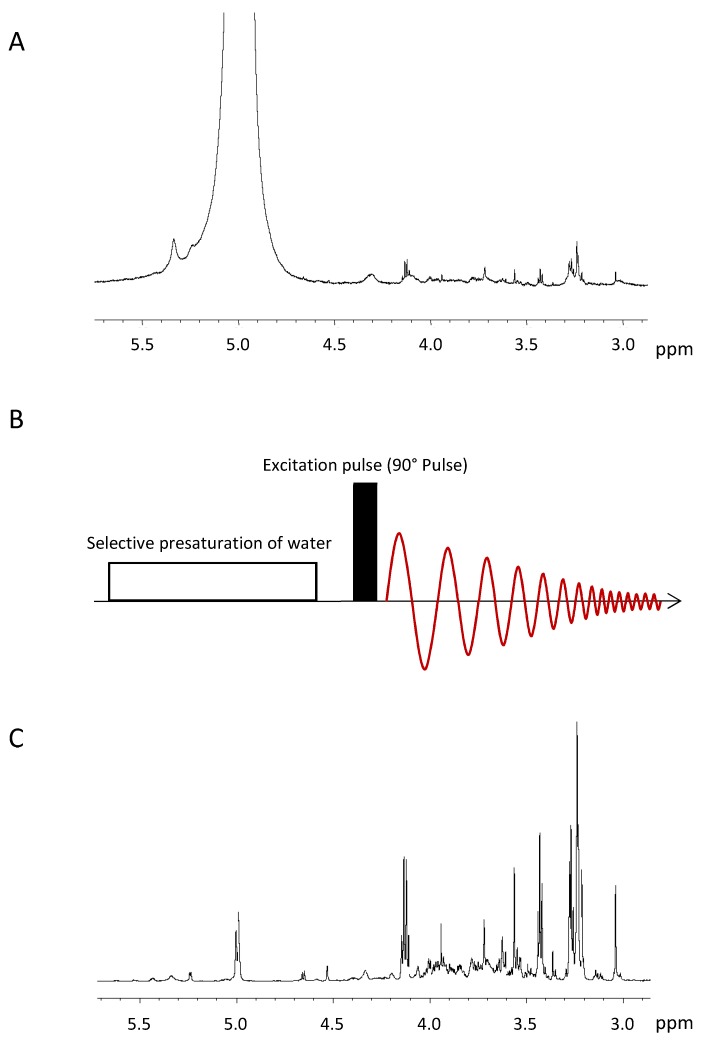
**(A)**^1^H NMR spectrum of a tumour sample without water suppression. The strong water signal (0 ppm) saturates the analogue to digital converter and leaves just a few bits for the metabolite signals, which impedes the quantification of the latter. **(B)** Schematic representation of the presaturation pulse sequence [58], which consists of a selective presaturation at the water frequency, the excitation of the sample and data acquisition. **(C)** ^1^H NMR spectrum of a breast cancer specimen with an almost completely suppressed water signal at 5 ppm. As a result, the metabolite signals of the sample can be quantified.

Water is a dominating component of biological samples, as a solute or as a solvent. Because of the high concentration of water protons, the ^1^H-NMR signal of water exceeds the metabolite signals by several orders of magnitude. If not suppressed, the water signal will saturate the analogue to digital converter (ADC), that is, it uses the full dynamic range of the ADC, leaving just a few bits for the metabolite signals, which impedes a correct quantification of the latter. Moreover, the strong water signal distorts the baseline of the spectrum. The ^1^H-NMR spectrum of a sample with high water content is displayed in Figure 3A.

Presaturation is the most simple and most widely used technique to suppress solvent signals [58] and can be easily combined with most pulse sequences used in NMR. It uses an extended period of a weak continuous wave irradiation at the frequency of the water signal at 4.7 ppm (Figure 3B). This irradiation results in an equal population of the two energy levels of the water hydrogen spins. Hence, if spin-lattice relaxation is neglected, there is no longitudinal magnetization of the water protons and the subsequent excitation pulse exclusively excites the signals of the metabolites. The power level employed for the presaturation must be chosen to only saturate the water signal and no metabolite signal in its vicinity. The determination of the presaturation power level is performed once for every sample in a series of experiments, where the power level is incremented in small steps and the outcome of the presaturation is evaluated by the operator. This evaluation has to take into account the intensities of the water signal and of the metabolite signals in its vicinity, with and without presaturation, as well as recommendations of the manufacturer concerning the maximum power level. Another important factor for the successful presaturation is the homogeneity of the B_0_ field, because signal broadening caused by inhomogeneity cannot always be suppressed by presaturation [58]. Figure 3C shows the spectrum of the same sample as in Figure 3A but here the water signal is suppressed using the described presaturation technique. The intensity of the residual water signal now is at the same level as the metabolite signal intensities and does not impede the quantification of the metabolite signals.

### 3.2. Pulse Sequences for 1D-NMR

**Figure 4:**
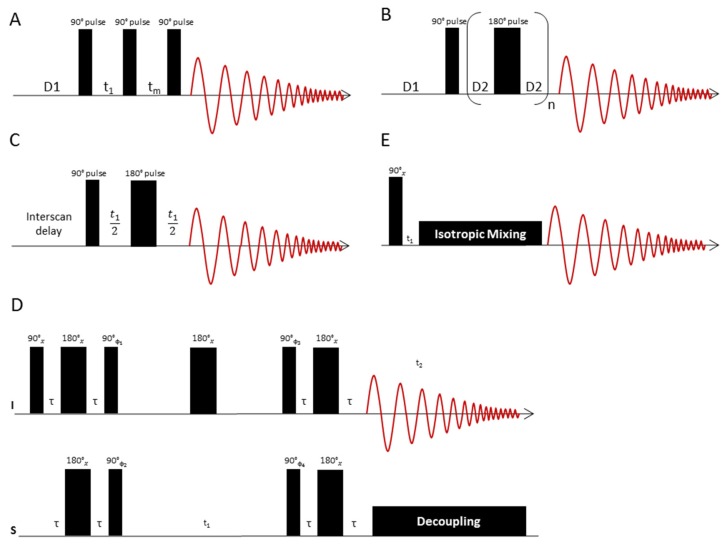
**(A)** The 1D NOESY pulse sequence consists of a presaturation interval (D1), an excitation pulse, which after a short time t_1_ (phase switching time) is followed by another 90° pulse. After another time t_m_, the spin system is excited with another 90° pulse. During the time t_m_, the water saturation is switched on again to ensure complete water suppression. D1 denotes the relaxation delay time. **(B)** Representation of the CPMG pulse sequence: After a presaturation interval (D1), the spin system is excited with a 90° pulse. This is followed by a train of n 180° pulses, each pulse bracketed by two delay times D2, in which the spins refocus. Subsequently, the signal is recorded as an FID. The total delay time n*τ after n spin echo periods τ = (D2-180°-D2) is chosen so as to suppress the signals of fast relaxing molecules like lipids. **(C)** JRES pulse sequence to separate ^1^H chemical shifts and ^1^H,^1^H couplings into separate dimensions of a 2D display. The chemical shifts are displayed in the horizontal dimension, while the multiplet patterns show up in the vertical dimension. If strong coupling artefacts can be neglected, the horizontal dimension corresponds to a “broadband” decoupled proton spectrum. **(D)** HSQC pulse sequence to correlate proton and carbon chemical shift information. The sequence starts with an INEPT block, which transfers proton magnetization to ^13^C. The carbon magnetization is then labelled with ^13^C chemical shift information via a spin echo sequence, the duration of which is incremented in subsequent experiments. A reverse INEPT transfer brings back ^13^C magnetization to the proton channel, where it is recorded under ^13^C broadband decoupling. **(E)** TOCSY pulse sequence to correlate ^1^H chemical shifts that are part of a spin-spin coupling network. The sequence starts with proton magnetization, which is labelled with its precession frequency during t_1_. In a subsequent isotropic mixing step, a net magnetization transfer to coupled protons is performed. The extent of this magnetization transfer can be steered by adjustment of the length of the mixing delay. Values of 80 ms typically give spectra where magnetization has been transferred to all coupling partners of the excited spin, so that the whole spin system can be traced out. The signals of the coupling partners appear on horizontal lines in the spectrum, which eases analysis of metabolite spin systems.

The 1D Nuclear Overhauser-Effect Spectrometry (1D NOESY) technique is employed when the goal is to observe both the signals of low-molecular weight compounds (metabolites) and macromolecules (lipids, proteins) in a ^1^H-NMR spectrum [23]. This experiment is helpful in breast cancer studies to estimate the amount of lipids in relation to the metabolite content. The 1D NOESY pulse sequence [RD-90°-t1-90°-tm-90°-ACQ] (Figure 4A) [23] starts with a presaturation irradiation of the water signal during the relaxation delay time (English "relaxation delay"—RD). The first 90° pulse hence produces transverse magnetization only for the metabolites, since the signal of water has previously been saturated. This is followed by a short interval t_1_ of approximately 3 μs, which serves as a switching time for the phases of the pulses. The subsequent second 90° pulse rotates the magnetization of the metabolites to the z-direction. During the time t_m_ (duration approximately 10 ms) after the second 90° pulse, presaturation is switched on again to once more saturate the water magnetization that has relaxed during the course of the experiment. The metabolite magnetization is not affected by this saturation because it is longitudinal at this time. Finally, the third 90° pulse rotates the magnetization of the metabolites back to the transverse plane. During the acquisition time (here denoted ACQ) the transverse magnetization is sampled. The magnetization of the water is saturated at this time and therefore provides no signal after the third 90° pulse.

If the aim is to observe the metabolite signals only, while the macromolecular background of lipids and proteins is suppressed, the Carr-Purcell Meiboom-Gill (CPMG) sequence is employed. This sequence, like the 1D NOESY sequence, has an integrated one-dimensional water presaturation interval. It uses the pulse train [RD-90 °-(τ1-180°-τ1)n-ACQ] (Figure 4B) [23], where (τ1-180°-τ1)n acts as a T_2_ filter to suppress signals from macromolecules and other substances with short T_2_ times. As a result, the ^1^H-NMR spectrum of tissue samples obtained with this pulse sequence only consists of small molecule signals from metabolites, which have relatively long T_2_ times. After presaturation, the excitation starts with a 90° pulse, which generates transverse magnetization. The subsequent precession of the magnetization during the delay time D2 (typically 1 ms for CPMG) is refocused by a 180° pulse followed by another delay D2. This spin-echo sandwich is repeated n times, followed by the acquisition after the n-th echo [23]. If the lipid content of the tissue is high, the echo time n*τ can be prolonged to suppress the lipid signals by spin-spin relaxation. Depending on the type of tissue, the total echo time can be between 30 ms [59] and 720 ms [60]. For some tissues, such as brain tumours, which have lower lipid content, the echo times are short and only vary between 30 and 150 ms [59,61]. Tissue with higher lipid content requires longer echo times to suppress the lipids. In breast cancer samples echo times as long as 580 ms have been used [24,60]. The CPMG pulse sequence is an indispensable tool in NMR studies of breast cancer tissue, as lipid signal suppression is essential.

Finally, it should be noted that the above-mentioned pulse techniques were originally designed for liquid state NMR measurements. When applied to HR-MAS NMR, the timing of the pulse sequence must be synchronized with the rotation period, so that the interpulse spacings are equal to multiples of the rotor cycle time [52].

### 3.3. Pulse Sequences for 2D-NMR

In the presence of strong peak overlaps, which are typical for complex mixtures such as those encountered in metabolomics, special measurement techniques are required to untangle the overlapping peaks and to assist in peak assignment. Peaks that overlap in the 1D NMR spectra can often be resolved in two dimensional (2D) NMR spectra.

Two dimensional J-resolved (2D JRES) NMR spectroscopy (Figure 4C) [62] is helpful for the analysis of metabolite mixtures, as it allows the recording of a second spectral dimension with relatively little overlap of signals. In the 2D JRES NMR experiment, the ^1^H spectrum is presented in the horizontal dimension and the coupling pattern of each signal is displayed in the vertical dimension. The number of identifiable metabolites is, however, strongly limited by the 2D JRES spectral resolution as well as by strong coupling effects, which hamper the applicability of this method especially at low field strengths.

The use of 2D ^1^H-^13^C heteronuclear NMR (Figure 4D) in metabolomics may be advisable to identify metabolites if the ^1^H-NMR spectrum is heavily congested. Among the 2D NMR methods, the 2D Heteronuclear Single Quantum Coherence (HSQC) technique [63] offers a very high resolution by incorporation of ^13^C chemical shift information. The HSQC pulse sequence correlates proton and carbon chemical shifts. The sequence starts with an Insensitive Nuclei Enhanced by Polarization Transfer (INEPT) block [64], which performs a polarization transfer of proton magnetization to the ^13^C channel. In a subsequent spin echo sequence, the carbon magnetization is labelled with ^13^C chemical shift information. The duration of the spin echo sequence is incremented in subsequent experiments. Finally, a reverse INEPT transfer brings back ^13^C magnetization to the proton channel. During the acquisition, ^13^C broadband decoupling is switched on to collapse the ^13^C, ^1^H-couplings. In many cases, however, HSQC is generally not sensitive enough for metabolomics studies but the acquisition of an HSQC spectrum is of particular importance if new metabolites have to be identified or if additional evidence is sought for signal assignments obtained from ^1^H-NMR measurements, as exemplified for breast cancer tissue in Reference [35].

A disadvantage of both HSQC- and of 2D-JRES spectra is the missing spin system information, as the cross-peaks are all independent of each other. This shortcoming is avoided by the 2D ^1^H-^1^H Total Coherence Spectroscopy (TOCSY) experiment [65], which permits the identification of individual ^1^H spin systems that can be assigned to the various mixture components. The TOCSY pulse sequence (Figure 4E) correlates distinct ^1^H-NMR signals, which are part of a network of spin-spin couplings (usually denoted as a “spin system”). An excitation pulse creates proton magnetization that is labelled with its precession frequency during the delay time t_1_, which is incremented in subsequent experiments. During an isotropic mixing step, a net magnetization transfer to coupled protons occurs, the extent of which is controlled by adjustment of the length of the mixing delay. Values of 80 ms typically give spectra where magnetization has been transferred to all coupling partners of the excited spin, so that the whole spin system can be traced out. The signals of the coupling partners appear on horizontal lines in the spectrum. TOCSY is particularly well suited for computational analysis, since each cross-section of signals represents the 1D spectrum of the whole spin system, that is, the signals of a metabolite. Notably, for HR-MAS NMR, adiabatic mixing sequences [66] are recommended to perform the isotropic mixing. The composite pulse mixing sequences commonly employed in liquid state NMR introduce modulations of the effective field in the presence of both radiofrequency field and B_0_ inhomogeneities, when applied to rotating samples [66]. These modulations compromise the performance of composite pulse mixing sequences and introduce a sensitivity of the signal intensities to the sample spinning speed. Adiabatic mixing sequences are less susceptible to such modulations and perform better [66].

As a final point, 2D NMR techniques that use pulse sequences adopted from liquid state NMR require a rotor synchronization [67], that is, all delays used in the pulse sequence must be integer multiples of the rotor period. Rotor synchronization helps to eliminate both residual anisotropic interactions and the effect of radial inhomogeneities of the radiofrequency field. Rotor synchronization is of particular importance for TOCSY experiments, where the lengths of every basic cycle of the isotropic mixing sequence as well as the trim pulses have to be integer multiples of the rotor period [67].

## 4. Metabolites Identified with HR-MAS NMR in Breast Tumour Tissue

Two complementary strategies are used in metabolomics: the targeted and the non-targeted approach. With a targeted approach, a preselected subset of metabolites is measured, usually based on an a priori hypothesis. Contrarily, with a non-targeted approach, the number of metabolites to be measured is not predetermined; rather the aim is to capture as much information as possible. To date, 27 publications using HR-MAS NMR spectroscopy report a total of 46 metabolites to be detectable in breast cancer tissue (tumour tissue from patients and/or xenografts) (Table 1). Cheng et al. published the first report using HR-MAS NMR to analyse breast cancer tissue already in 1998 [49], followed by the milestone work of Sitter et al., who identified more than 30 metabolites, in 2002 [24]; however, no quantification was performed in these early studies. Table 1 gives an overview of metabolites detected in breast cancer tissue analysed by HR-MAS ^1^H-NMR spectroscopy and in addition indicates if metabolite concentrations were determined, for example, in µmol/g tissue, using an internal- (TSP) or external standard (ERETIC, PULCON) (twelve publications) or if relative quantification based on integrated peak areas was performed (eight publications).

The most comprehensive quantitative investigations of breast cancer tissue using HR-MAS NMR were those by Park et al. and Yoon et al., where 34 metabolites were reported in each study [50,68]. The exact inclusion criterion for HR-MAS NMR-based identification of metabolites in breast cancer tissue is, however, rarely stated in the literature. In the work of Sitter et al., reporting concentrations for nine metabolites, a signal-to-noise ratio (SNR) >10 for the creatine singlet was applied [41]. In a recent publication, we quantified all metabolites that had a baseline-separated signal with a SNR >3 for at least one peak used for quantification, thus reporting concentrations for 32 metabolites [35].

As indicated in Table 1, the quantification of approximately 40 metabolites is what can be maximally achieved by this method in breast cancer tissue with a non-targeted approach. A substantial further increase of the number of metabolites to be quantified would require higher B_0_ field strengths at the expense, however, of proportionally increasing rotation speeds to shift the spinning sidebands out of the spectral window. The concomitant increase in centrifugal forces acting on the fragile samples would compromise the non-destructive nature of NMR. Currently, HR-MAS spectra are acquired at a maximum ^1^H frequency of 600 MHz (14.1 T), which gives sideband-free spectra at a rotation speed of 5 kHz. There are, however, approaches to use slow-spinning HR-MAS techniques [55] that employ a sideband suppression like PASS [69], PHORMAT [70] or PROJECT [71], which suggest that high quality HR-MAS spectra could be obtained at higher field strengths than 14.1 T. However, the application of PASS and PHORMAT to tissue analysis is hampered, not only by sensitivity and resolution problems but especially by the fact that the extent of the side band pattern affects the intensity of the isotropic peak. This feature impedes quantification, as the extent of the sideband pattern may vary from sample to sample [55]. A suitable approach to slow MAS in metabolomics seems to be the PROJECT pulse sequence but so far spectra devoid of spinning sidebands were only observed at 400 Hz rotation speed in case of favourable conditions, such as a spherical sample (for instance fish eggs), minimal B_1_ inhomogeneities, small-volume rotors and a sample composition close to that of an isotropic liquid [55].

Finally, the quantification of metabolites from 1D ^1^H-NMR can be difficult due to the complexity of the spectrum. For instance, in 1D HR-MAS ^1^H-NMR spectra, choline- and ethanolamine-containing metabolite signals are superimposed and the weak signals from phosphoethanolamine and glycerophosphoethanolamine are difficult to detect. HR-MAS ^31^P-NMR spectra have higher chemical shift dispersion than their ^1^H-NMR counterparts, which helps to separate the signals of choline- and ethanolamine-containing metabolites. The quantification of the signals in the HR-MAS ^31^P-NMR spectra is fairly straightforward, as the signal of phosphocholine shows up in both types of spectra (^1^H and ^31^P) and can be used for cross-calibration. Glycerophosphoethanolamine and glycerol-3-phosphate were only reported in breast cancer tissue in the one study that used HR-MAS ^31^P-NMR [72].

## 5. Metabolite Quantification with HR-MAS NMR

There are several options for quantitative analysis with HR-MAS NMR spectroscopy, which have certain advantages and disadvantages. As already mentioned above, the method for absolute quantification used in twelve publications to determine metabolite concentrations in breast cancer tissue is indicated in Table 1.

**Figure 5:**
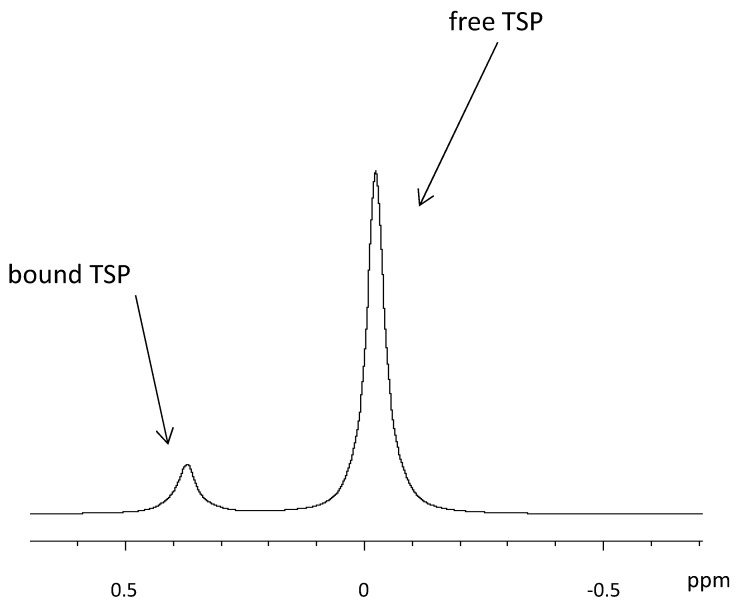
Influence of TSP interaction with tissue on the quantification with ^1^H HR-MAS NMR spectroscopy.

One approach is the quantification with an internal standard, which is at the same time used for calibrating the spectrum [39]. Tetramethylsilane (TMS) is widely used as internal standard in organic chemistry. However, it is insoluble in water and therefore its water-soluble forms, trimethylsilylpropionate (TSP) [73] and sodium 2,2-dimethyl-2-silapentane-5-sulfonate (DSS) are used in HR-MAS NMR [74]. As the weaker acid compared to DSS, TSP is more affected by sample pH. Both TSP and DSS are reported to bind to hydrophobic parts of proteins [75], which partly distorts the quantification. In prostate tissue, it was observed that more than 70% of the added TSP became “NMR-invisible,” because the TSP was bound to macromolecules in the tissue [76]. If the TSP signal is subsequently used for quantification, the observed error in the TSP peak area gives rise to a significant over-estimation of the metabolite concentrations, as was also mentioned elsewhere [73]. In their tutorial about NMR metabolomics analysis [77], the authors state that large deviations of the TSP peak area and shape in a series of samples are often due to an insufficient suppression of macromolecules like proteins, as they are partners for non-specific bonding to TSP and DSS. Nowick et al. suggested DSA (4,4-dimethyl-4-silapentane-1-ammonium trifluoroacetate) as a new internal standard that does not suffer from interactions with cationic peptides like DSS [78]. Alum et al. compared DSA to TSP and found that the integral of the DSA signal correlated linearly with its concentration under all pH, whereas no such linear correlation could be found with TSP. The authors suggest DSA to be used both as a universal chemical shift reference and a concentration standard [79]. In Figure 5, examples of free and breast cancer tissue-bound TSP are shown. It is also possible to use the water content of the tissue as an internal standard [19,60], as demonstrated on brain tissue samples [80]. However, it is questionable whether quantification based on water content is well suited in heterogeneous tissue types, such as breast cancer, where fat and therefore also water content varies greatly.

External standards are also used for metabolite quantification with HR-MAS NMR. A widely used method to quantify metabolites in tissue is ERETIC (Electronic REference To access In vivo Concentrations), an artificially generated radio frequency signal, which is pre-calibrated to a reference sample, such as sucrose, in an independent measurement [81,82]. The advantage of ERETIC is that no standard has to be added to the sample and thus no distortion due to the interaction between the standard and the sample matrix can occur. This was shown for instance by Martinez-Bisbal et al., who compared metabolite concentrations obtained with ERETIC and the internal standard DSS in tissue biopsies from glioblastoma multiforme, observing consistently higher concentrations with DSS [74]. Sitter et al. found a larger relative standard deviation (RSD) with ERETIC (> 6.7%) than with TSP (> 4.4%) for the quantification of serial dilutions of creatine in phosphate buffered saline (1, 5 and 10 mM) [39], which they attributed to radiofrequency inhomogeneity influencing ERETIC more than TSP. Nevertheless, the authors still recommended ERETIC as the better alternative, since this method avoids matrix effects, is stable, accurate and precise and has a reproducible signal area, as reported by Albers et al. [76]. To assess the robustness of ERETIC- and TSP based quantification, Albers et al. studied the stability of their peak areas in solution over time, reporting a long-term RSD of 4.10% for ERETIC and 2.60% for TSP. An external standard other than ERETIC has been used by Taylor et al. [52]. Here, the authors applied a silicone rubber sample to function as an external standard for both frequency reference (0.06 ppm from TMS) and quantification. The rubber sample (approximately 100 μg) was permanently mounted inside a Kel-F spacer in the MAS rotor in such a way that it is not in contact with the sample but is located inside the detection coil. Pulse length-based concentration determination (PULCON) can also be used to measure the concentrations in 1D NMR spectra [83], especially when several (unknown) signals are superimposed. This methodology was originally developed for the analysis of proteins. The sequence consists of first determining the 360° pulse after tuning and matching. After the integration of known signals, the protein concentration can be determined [83]. This is particularly helpful if one or more superimposed signals are unknown. PULCON is rarely used in HR-MAS NMR-based metabolomics and its use in breast cancer metabolomics is reported only once [42]. In view of the high technical reproducibility and the fact that ERETIC is free from matrix effects [76], ERETIC is today generally considered the preferred method for metabolite quantification in tissue by means of HR-MAS NMR spectroscopy. Nevertheless, since the performance of different methods for quantitative determination of metabolites in tissue by means of HR-MAS NMR has not yet been directly compared, a comprehensive study of the above-mentioned methods would be of great advantage.

One potential problem with metabolite quantification in breast cancer tissue with HR-MAS NMR is related to the presence of strong lipid signals that cannot be sufficiently suppressed by the CPMG pulse sequence. This has been reported to particularly affect the quantification of lactate, since the lactate methyl resonance at 1.32 ppm may be masked by a broad lipid signal in lipid-rich environments [38,84]. Even though the quantification of lactate was possible in several other studies [39,50,68,85], the detection of lactate in tissue may require a selective excitation technique, such as the Sel-MQC sequence of He et al., which is a spectral editing sequence that uses multiple-quantum filtration [86]. However, experimental problems, such as B_1_ inhomogeneity, are a challenge for the reliable quantification of low lactate concentrations [87] and, in addition, severe signal losses can lead to inefficient suppression of unwanted lipid signals. Therefore, an improved spectral editing scheme that is robust to inhomogeneous fields was recently reported and shown to achieve selective excitation of lactate with minimal signal loss [87,88]. In addition, a variant of this pulse sequence [88] provides in-phase magnetization, which can be more accurately quantified than the antiphase magnetization of the pulse sequence [86] and allows the selection of experimental parameters that meet optimal lipid suppression requirements [88]. Lactate editing was originally developed for MRS [88] but it has been demonstrated that it can also be used in HR-MAS ^1^H-NMR to quantify lactate in intact lipid-rich tissue [36]. This approach can, in theory, be extended to other metabolites, that can be edited employing multiple-quantum filtration techniques. This holds especially for alanine and threonine [87]. In summary, sophisticated spectral editing techniques that are based on Optimal Control (OC) theory [89] have become available, that allow for the design of tailored pulses that excite only the signal of a given metabolite. However, these have so far merely been applied to the quantification of lactate and alanine [87].

The problem with superimposed signals is not restricted to that of strong lipid signals. The "add to subtract" approach, described by Ye et al. [90], is one approach to handle superimposed signals. Here, the signals of metabolites which are usually strong like lactate or glucose, are subtracted from the spectrum prior to analysis and small, low-concentration molecules, which may be of biological significance, appear and can be quantified [8]. Another approach to deconvolute superimposed signals, which has so far only been used in in vivo NMR, is the Linear Combination of Model (LCModel) method [91,92]. This method analyses an NMR spectrum as a linear combination of model spectra obtained from individual metabolite solutions, using a constrained regularization method which takes the baseline and the line shape of the spectra into account without employing a restrictive parameterization on the data [91,92]. Using LCModel analysis, a set of in vitro metabolite spectra combined with simulated lipid and protein spectra assists in the analysis of HR-MAS spectra. Application of this LCModel setup to brain tumour biopsy HR-MAS data revealed interactions between metabolites and the macromolecular background via the analysis of small peak shifts [28]. Moreover Opstad et al showed, that LCModel provides a user-independent protocol of analysis of brain tumour HR-MAS spectra [28]. So far, however, no applications of LCModel analysis to breast cancer tissue HR-MAS spectra have been reported.

**Figure 6:**
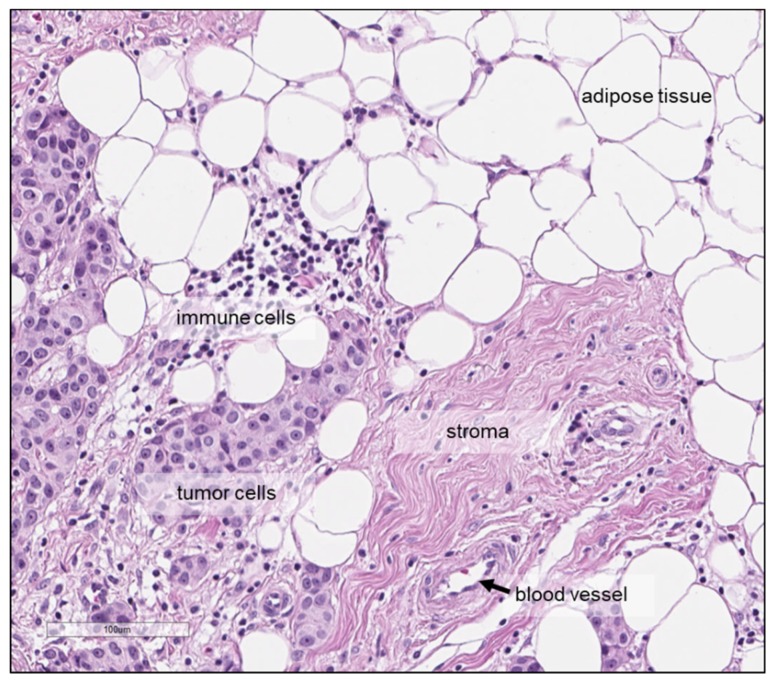
Haematoxylin-eosin staining showing the tumour tissue microenvironment of a breast carcinoma and different types of cells, including tumour cells, immune cells and adipocytes—as well as stroma and blood vessel. Scale bar: 100 μm.

Finally, a factor which is independent of the quantification method but that may affect the quantitative analysis when metabolite concentrations are determined per gram tissue, as is commonly done with HR-MAS NMR, is related to the cellular composition of the tumour tissue specimen, which might vary within a tumour as well as between tumours. Tumour tissue does not only consist of tumour cells but also of, for example, cancer-associated fibroblasts, endothelial and lymphatic cells and cells of the immune system. Moreover, adipocytes may be present in breast carcinomas (Figure 6) and breast tumour tissues also differ with regard to the amount of tumour cells in relation to the amount of necrosis and extra-cellular matrix-rich stroma. Consequently, tissue samples from two different tumours or samples from two different areas within the same tumour, with the same weight may have different cellular content, which may translate into differences in metabolite content. Several studies assessed the tumour cell content in haematoxylin-eosin stained tissues sections, either prepared after HR-MAS NMR or from an adjacent tumour area, with notable differences between the tumours [84,93]. Some authors reported study inclusion criteria based on the tissue composition; for instance, >30 tumour cells [38] or >5% tumour area [85] were used as an inclusion criterion, while others excluded samples with a high lipid content [43]. The influence of the tissue composition on the observed metabolite levels should be taken into account, since correlations between metabolite concentrations and tissue composition have been reported, including higher levels of glycine, GPC and phosphocholine with higher tumour cell fraction [39], as well as poor SNR in spectra with a high level of connective tissue [93].

Intra-tumour variability of metabolite concentrations may, in addition to differences in the tissue composition, be related to the coexistence of different subpopulations of cancer cells, which differ in their genetic and phenotypic characteristics [94,95]. Therefore, despite the high technical reproducibility of NMR [8], recognizing the potential impact of intra-tumour heterogeneity on metabolite concentrations determined by quantitative HR-MAS NMR is essential. Understanding intra-tumour heterogeneity is also a prerequisite to decide whether analysis of a small number of samples or even a single biopsy, is sufficient to obtain information representative for the entire tumour. Thus far, three studies addressed intra-tumour differences in metabolite concentrations in breast tumour tissue [35,50,96]. Based on the correlation between paired samples from the same tumour compared to random sample pairs, Cao et al. stated that the metabolic profile varied more between the different tumours than within the tumours [96]. This conclusion is supported by a second investigation, where intra-tumour concentration differences were assessed for 32 metabolites, as well as lipid content indicated by the signals at 1.3 ppm and 0.9 ppm, by sampling 8-10 tissue cores (diameter: 2 mm) from resected breast tumour tissue and duplicate cores from additionally 15 breast tumours [35]. A high degree of intra-specimen variability was observed in the tumour tissue (mean RSD: 0.48-0.74) compared to normal liver tissue (mean RSD: 0.16-0.20), which is morphologically more homogeneous. Nevertheless, it was shown that inter-tumour differences were, on average, larger than those observed within a tumour, suggesting that the analysis of one or a few, replicates per tumour might be sufficient. Park et al. used a slightly different approach and considered also the intra-tumoural localization by sampling tissue cores from both the tumour centre and periphery from surgically removed tumour tissue, concluding that the intra-tumoral localization had a limited impact on the observed concentrations of the 34 reported metabolites [50].

## 6. Significant Associations with Clinical Factors

**Figure 7:**
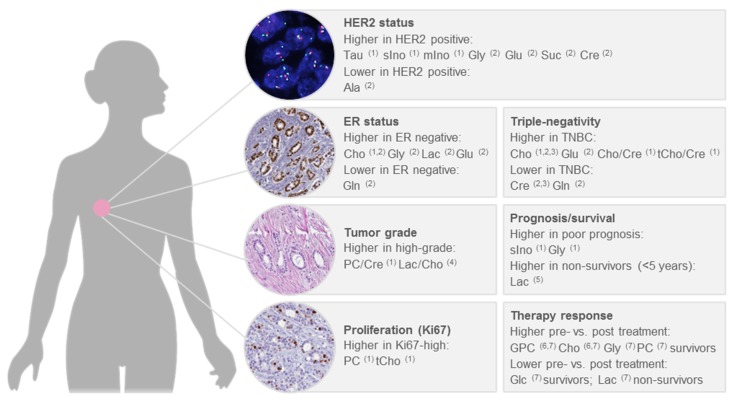
Significant associations of metabolite levels determined by HR-MAS NMR with clinicopathological factors in human breast cancer. ^1^ Choi et al. 2012 [47]; ^2^ Cao et al. 2014 [96]; ^3^ Moestue et al. 2010 [40]; ^4^ Cheng et al. 1998 [49]; ^5^ Giskeödegård et al. 2012 [85]; ^6^ Cao et al. 2012b [38]; ^7^ Cao et al. 2012a [37].

Breast cancer is a heterogeneous disease, with diverse biological features as well as clinical behaviour. To assess the clinical course of the disease and to make decisions about treatment, factors such as tumour stage, tumour grade, oestrogen receptor (ER) and progesterone receptor (PR) status and expression of the human epidermal growth factor receptor (HER2) are considered. Chemotherapy is indicated for tumours that are negative for ER, PR and HER2 (triple-negative) and also represents the only available therapy for this subtype, in addition to surgery [97]. For the HER2 positive subtype, anti-HER2 therapy and chemotherapy are recommended, irrespective of ER status; whereas, hormonal therapy is recommended for ER positive tumours, with chemotherapy additionally administered in case of a high risk of recurrence, as indicated by tumour grade, proliferation or a prognostic gene expression assay [97]. Several studies thus far used quantitative HR-MAS NMR to correlate metabolite levels in intact breast cancer tissue with clinicopathological factors [39,40,41,47,96], survival [37,38] or treatment response [38,48]. The main findings of these studies are summarized below, focusing on significant differences of metabolite levels between clinically relevant patient subgroups (Figure 7).

Significantly higher choline concentrations in ER- and PR- tumours, as well as higher concentrations of creatine and taurine in PR- tumours, were reported by Choi et al. [47]. An elevated level of choline in the ER- subtype was also reported by Cao et al., together with higher levels of glycine, lactate and glutamate and lower levels of glutamine [96]. In relation to HER2 positivity, significant associations with higher levels of taurine, scyllo-inositol and myo-inositol [47], as well as higher levels of glycine, glutamine, succinate, creatine and lower levels of alanine [96], were described. Triple-negative status was found to be associated with higher choline levels in three separate publications [40,47,96] and with lower creatine levels in two publications [40,96]. Moreover, triple-negativity was associated with higher choline to creatine and total choline to creatine ratios [47], higher levels of glutamate and lower levels of glutamine [96]. Comparing basal-like and luminal-like xenografts, glycerophosphocholine and glycine were higher in the basal-like and phosphocholine was higher in the luminal-like [40].

Few studies thus far looked at metabolite concentrations in relation to tumour grade. A significantly increased phosphocholine to creatine ratio in grade III compared to grade I-II tumours was reported by Choi et al. and higher concentrations of phosphocholine and total choline were reported by the same authors in highly proliferative tumours, as assessed by Ki-67 [47]. The association between phosphocholine and tumour grade is supported by findings from the first study of metabolites in breast cancer tissue using HR-MAS NMR, where Cheng et al. showed a higher phosphocholine to choline ratio in high-grade tumours already in 1998, although this difference was not statistically significant [49]. A significantly higher lactate to choline ratio in high-grade tumours was also reported [49].

Sitter et al. observed higher concentrations of choline and glycine in tumours larger than 2 cm [41]. Taking several clinicopathological factors into account and comparing tumours from patients with good prognosis (defined as node-negative, < 2 cm and ER+ and PR+) and poor prognosis (node-positive, > 2 cm or ER- or PR-), significantly higher concentrations of scyllo-inositol and glycine were characteristic for the tumours from patients with poor prognosis [47]. Another study identified no significant differences between prognostic groups, defined similarly, for individual metabolites; however, a trend towards higher concentrations of glycine in tumours from patients with a poor prognosis was observed [39]. Using survival time shorter or longer than five years as the endpoint, higher lactate levels were found in ER+ non-survivors, while higher levels of glycine almost reached statistical significance [85]. The association of lactate and glycine with survival is supported by a second study, although here the reported association were not statistically significant [38]. Whereas we here focused primarily on studies that used quantitative HR-MAS NMR to correlate metabolite concentrations with clinicopathological factors, the use of multivariate modelling for prediction of factors, such as tumour grade, oestrogen receptor status and lymph-node status, has also been pursued [46,98]. Future studies of larger patient cohorts, with accompanying information on metastasis-free and overall survival, are warranted to clarify if metabolic signatures can be used to predict clinically relevant prognostic endpoints, such as identifying patients with a low risk of disease recurrence.

The response to neoadjuvant therapy also provides information on prognosis, since a pathological complete response (pCR) is indicative of a lower risk of recurrent disease [99]. Comparing patients with a complete pathological response (pCR) after neoadjuvant anthracycline and/or taxane-based chemotherapy to patients who did not achieve a pCR, no significant differences in metabolite concentrations were found; only, a trend towards a lower phosphocholine to creatine ratio in patients with pCR was observed [48]. In a recent study by Euceda et al., including 122 breast cancer patients with biopsies taken before, during and after neoadjuvant 5-fluorouracil, epirubicin and cyclophosphamide, followed by taxane-based therapy – where the patients were additionally randomized to receive bevacizumab or not – principal component analysis (PCA) indicated overall changes in the metabolic profile with chemotherapy over time [43]. However, no metabolic differences were found between pre-treatment biopsies from pathological complete responders and non-responders. Moreover, no significant differences in metabolite levels were found between patients treated with chemotherapy only and patients treated with chemotherapy plus bevacizumab, either before, during or after completion of therapy [43]. In a study of triple-negative patient-derived xenografts, the response to everolimus, an inhibitor of mammalian target of rapamycin (mTOR), could not be predicted based on the metabolic profile [44]. The prediction of chemotherapy response prior to treatment thus appears to be difficult based on the metabolic evaluation of pre-treatment biopsies. The observed differences between the metabolic profile before and after treatment can be further explored to reveal mechanisms behind therapy response and resistance but may also reflect differences between tumour and normal breast tissue. Analysis of changes in metabolite levels between 33 paired pre- and post-treatment specimens showed lower glycerophosphocholine and choline levels post-treatment compared to pre-treatment in survivors (≥ 5 years), while no significant differences pre- versus post-treatment were found in non-survivors (< 5 years) [38]. In another study of the same authors, where tumour tissue specimens from 89 breast cancer patients who received neoadjuvant chemotherapy were analysed, survivors showed a significant decrease in the levels of glycine, choline, phosphocholine and glycerophosphocholine and an increase in glucose, post-treatment compared to pre-treatment; whereas, non-survivors displayed an increased level of lactate after treatment [37]. A different metabolic response to PI3K/mTOR inhibition between basal-like and luminal-like patient-derived xenografts was also reported, with lower level of phosphoethanolamine and higher levels of phosphocholine and glycerophosphocholine compared to untreated controls in basal-like but not in luminal-like, xenografts [72]. This study also highlights the potential of using ^31^P HR-MAS NMR in biomarker studies to analyse phosphorus-containing metabolites, such as phosphoethanolamine, for which the weak signal may be difficult to detect using ^1^H HR-MAS NMR.

## 7. Summary

This review provides an overview of analytical aspects related to the use of quantitative HR-MAS NMR for metabolic profiling of intact breast tumour tissue, including an overview of the most widely used NMR techniques and briefly summarizes significant findings with regard to metabolite levels and clinically relevant factors.

A robust and standardized protocol is required for reproducible analysis of metabolite concentrations in tumour tissue samples from large patient cohorts. This means that factors such as the time and the temperature of each step — from sample collection during surgery to the actual measurement — must be taken into account. Although tissue freezing can alter the metabolic profile, there is often no better alternative since the logistics needed for the direct analysis of fresh tissue after surgery is missing, making snap-freezing of the tissue, followed by storage at −80 °C, indispensable. A simple rule of thumb is to minimize the time period before freezing, followed by storage in a low-temperature freezer. During the measurement, a low temperature, for example, 4 °C, for a time as short as possible, might be preferable but the effect of these factors on the observed metabolite concentrations has not been directly studied in breast cancer tissue thus far. Clearly, a frozen sample would be inappropriate for HR-MAS NMR because of the associated line broadening. A too strong reduction of the measurement time would make the identification of certain metabolites impossible. Finally, no direct comparison has been made to study the impact of rotation frequency on metabolite concentrations in breast cancer tissue.

After the generation of the HR-MAS NMR spectrum, the correct identification and quantification of metabolites is crucial. The limited resolution and sensitivity of the method is reflected in the number of specific metabolites and in the analytical accuracy, which can be obtained. Typically, approximately 40 metabolites in tissue can be determined by HR-MAS NMR. Problems of spectral overlap can be alleviated by using higher magnetic field strengths, which improves both sensitivity and resolution. Another approach to improve accuracy is to cope with the overlap problem by decoupling ^1^H-^1^H- scalar interactions via pure shift methods [100] which give peaks free of splittings. These techniques suffer, however, from sensitivity losses and are merely suitable for the profiling of high concentration metabolites. Another boost of accuracy can be obtained from hybrid techniques like the combination of MS/MS and NMR [100]. In the presence of strong peak overlaps and especially if previously unknown metabolites have to be identified, signal assignment may require assistance by 2D NMR techniques. Here, TOCSY is particularly valuable since it shows which peaks belong to a shared spin system and thus to a metabolite. In addition, HSQC provides information about the *^1^J* correlation of ^1^H NMR signals with ^13^C nuclei. By combining these two methods, it is possible to assign a particular spin system from the ^1^H NMR spectrum to a metabolite and unambiguously identify it by means of its ^13^C NMR shifts. Still, since tissue concentrations of many metabolites are very low, identification may be problematic. This may be compensated by increasing the measurement time in 2D-NMR studies. However, increasing the measurement time will at some point be insufficient, since the slope of the SNR can only be improved by exponentially increasing the number of scans, eventually making further improvements infeasible.

Following metabolite identification, the choice of the quantification method is also important. Nowadays, ERETIC has become established as the method of choice, since it avoids the protein binding observed with internal standards such as TSP. However, for absolute quantification further aspects must be taken into account. In most publications that used HR-MAS NMR for quantification of metabolites in breast cancer tissue, the sum of the acquisition time and the relaxation delay was greater than 5 × T_1max_, where T_1max_ denotes the spin-lattice relaxation time of the slowest-relaxing metabolite. Therefore, a correction of the effects of T_1_ relaxation is not necessary for quantification. On the other hand, to achieve absolute concentrations, a T_2_ correction has to be applied to compensate for the signal loss during the extended echo train. Most of the publications listed in Table 1 used spin-echo sequences with approximate total durations of 285 ms but did not report a T_2_ correction, that is, T_2_ relaxation losses were deliberately accepted. Since the determination of T_2_ times is not trivial due to the low intensity of some metabolites and the high fat content in some breast cancer specimens, T_2_ time determination in HR-MAS NMR based metabolomics is a matter of further investigations. However, for many purposes, such as the comparison of metabolite levels between clinically relevant subgroups, relative concentrations are sufficient.

An important question is why one should use HR-MAS NMR for metabolic profiling of tissue when MS-based methods provide information about a considerably larger number of metabolites. The availability of human tissue for scientific research is limited and its collection is associated with great organizational and time effort. Therefore, in explorative tissue profiling studies, it is natural to opt for an approach that obtains as much information as possible from a limited amount of tissue. The detection and quantification of up to 46 metabolites in breast cancer tissue using HR-MAS NMR has been shown to be feasible by a number of groups (Table 1). Moreover, the non-destructive nature of HR-MAS NMR sets it aside from MS-based methods, since it allows the analysis of metabolites to be combined with other analytical techniques on the same tissue specimen. This may be important when small amounts of tissue are available, such as when working with pre-treatment core needle biopsies or resection specimens from early stage tumours, where most of the tissue is fixed in formalin and used to establish the pathological diagnosis. Histological examination after HR-MAS NMR has for instance been pursued [38,85], providing information about the tissue composition, such as the percentage of tumour cells. It has also been shown that high-quality RNA, with RNA integrity (RIN) values in the range 7–10, can be isolated from tissue after HR-MAS NMR [36,84]. Therefore, a combinatory approach with transcriptomics is feasible. Such a combination has for instance been used to identify subtypes of luminal-A breast cancers [84]; however, these subtypes are yet to be proven to have clinical relevance. A combination of HR-MAS NMR-based metabolomics, transcriptomics and proteomics—although using tissue from different areas of the tumour sample—has also been pursued. A recent study used hierarchical clustering based on metabolite data from a large breast cancer cohort generated by HR-MAS NMR to identify three metabolic clusters, with differences in glycerophospholipid metabolism and glycolysis [45]. Interestingly, these clusters neither overlap with classification based on grading, nodal status, tumour size or hormone receptor status, nor with the gene expression-based PAM50 subtypes. One cluster contained an overrepresentation of tumours with lobular histology as well as all ductal carcinomas in situ and significant differences between clusters were also found for the reactive I and II subtypes (Cancer Genome Atlas Network 2012) defined based on reverse phase protein array analysis (RPPA), as well as for genes related with extracellular matrix, basement membrane and cell adhesion. Whether these clusters are also linked to prognosis remains to be clarified. Generally, it remains to be determined whether metabolic biomarkers quantified in breasts cancer tissue ex vivo by HR-MAS NMR are useful in a clinical setting. Today, clinicopathological parameters are used in the clinic, together with commercially available gene expression-based assays, to predict the risk of recurrence. Several studies used quantitative HR-MAS NMR to study associations between metabolite concentrations in intact human breast cancer tissue with clinicopathologic factors and clinically relevant endpoints, such as survival or therapy response but relatively few statistically significant findings were reported that were additionally validated in independent studies. Small sample sizes and large intra-group variability in some cases likely contribute to the lack of statistical significance. Consistently, higher levels of choline-containing metabolites have been reported to be associated with poor prognostic features, including tumour grade, proliferation and ER negativity [47], triple-negativity [96] and a tumour size larger than 2 cm [41], as well as higher levels of glycine with larger tumour size [41] and survival time shorter than 5 years [85], although the latter did only reach borderline significance. Higher lactate levels in non-survivors were also reported in two separate studies [38,85], although statistical significance was only reached in one study [85].

In conclusion, additional studies with larger numbers of patients are required to establish reliable associations between metabolites and prognosis of breast cancer. Moreover, studies are required to analyse whether metabolites contribute information independent from transcriptomics. Nonetheless, a unique advantage of HR-MAS NMR is that tissue can be analysed in a non-destructive manner which allows combination of this technique with either transcriptomics or with other omics techniques.

## Figures and Tables

**Table 1:** Overview of metabolites reported in HR-MAS NMR breast cancer tissue studies. Q: absolute quantification with concentrations given in e.g. µmol/g tissue; R: relative quantification; I: identified but not quantified; ^#^patient tumour tissue; ^$^xenograft tissue; (A) ERETIC; (B) TSP; (C) PULCON.

	Gogiashvili et al. 2018 ^# (A)^	Tayyari et al. 2018 ^#^	Euceda et al. 2017a ^#^	Euceda et al. 2017b ^$^	Park et al. 2016 ^# (B)^	Yoon 2016 ^# (B)^	Haukaas 2016a ^$^	Haukaas 2016b ^#^	Chae 2016 ^# (B)^	Cao et al. 2014 ^#^	Grinde et al. 2014 ^$ (C)^	Choi et al. 2013 ^# (B)^	Borgan et al. 2013 ^$ (A)^	Bathen et al. 2013 ^#^	Giskeødegård et al. 2012 ^#^	Cao et al. 2012b ^#^	Cao et al. 2012a ^# (A)^	Choi et al. 2012 ^# (B)^	Li et al. 2011 ^#^	Sitter et al. 2010 ^# (A)^	Moestue et al. 2010 ^# $ (A)^	Borgan et al. 2010 ^#^	Giskeødegård et al. 2010 ^#^	Bathen et al. 2007 ^#^	Sitter et al. 2006 ^# (B)^	Sitter et al. 2002 ^#^	Cheng et al. 1998 ^#^
3-Hydroxybutyrate	Q																										
Acetate	Q				Q	Q		R				I		R												I	
Adipate					Q																						
Alanine	Q	R	R	R	Q	Q	R	R	Q	R		Q		R	I	I		Q	I			I	I			I	I
Arginine		R			Q	Q			I																		
Ascorbate	Q		R	R			R	R						R	I								I				
Asparagine					Q	Q			I																	I	
Aspartate	Q				Q	Q						I							I							I	I
ATP		R																									
Betaine	Q				Q	Q																			I		
Choline	Q	R	R	R	Q	Q	R	R	Q	R	Q	Q	Q	R	I	R	Q	Q	I	Q	Q	I	I	I	Q	I	I
Creatine	Q	R	R	R	Q	Q	R	R	Q	R	I	Q	Q	R	I	I	I	Q	I	Q	Q	I	I	I	Q	I	I
Ethanol						Q																					
Ethanolamine					Q	Q																					
Formate									I																		
Fumarate					Q	Q																					
Glucose	Q	R	R	R	Q	Q	R	R		I		I		R	I	R	I			Q		I		I	Q	I	
Glutamate	Q	R	R	R	Q	Q	R	R	I	R	I	I		R	I			I	I							I	I
Glutamine	Q	R	R	R	Q	Q	R	R	I	R	I			R	I											I	I
Glutathione	Q	R	R	R			R	R																			
Glycerol					Q	Q			I																		
Glycine	Q	R	R	R	Q	Q	R	R	Q	R	I	Q	Q	R	R	R	Q	Q	I	Q	Q	I	I	I	Q	I	I
GPC	Q		R	R	Q	Q	R	R	Q	I	Q	Q	Q	R	I	R	Q	Q		Q	Q	I	I	I	Q	I	
Histidine					Q	Q																				I	
Inosine	Q																									I	
Isoleucine	Q				Q	Q			I			I							I							I	
Lactate	Q	R	R	R	Q	Q	R	R	I	R	I	I	Q	R	R	R	I	I	I	Q		I	I	I	I	I	I
Leucine	Q	R			Q	Q			I			I										I				I	
Lysine	Q	R			Q	Q			I			I														I	I
Methionine	Q	R			Q	Q																					
*myo*-Inositol	Q	R	R	R	Q	Q	R	R	Q	I	I	Q		R				Q	I	Q		I		I	Q	I	
O-Phosphocholine	Q	R	R	R	Q	Q	R	R	Q	R	Q	Q	Q	R	I	R	Q	Q	I	Q	Q	I	I	I	Q	I	I
O-Phosphoethanolamine	Q				Q	Q			I		I														I	I	
Phenylalanine	Q	R			Q	Q			I																	I	
Proline	Q				Q	Q																					
*scyllo*-Inositol	Q			R				R		I				R				Q				I			I	I	
Serine	Q				Q	Q			I																		
Succinate	Q		R	R			R	R	Q	R		I						Q								I	
Taurine	Q	R	R	R	Q	Q	R	R	Q	I	I	Q	Q	R	I	R	Q	Q	I	Q	Q	I	I	I	Q	I	I
Threonine	Q	R			Q	Q																					
Tyrosine	Q	R	R	R	Q	Q	R	R	I																	I	
Uracil					Q	Q																				I	
Uridine		R																									
Valine	Q	R			Q	Q			I			I							I							I	I
